# The excretory-secretory products of *Echinococcus granulosus* protoscoleces stimulated IL-10 production in B cells via TLR-2 signaling

**DOI:** 10.1186/s12865-018-0267-7

**Published:** 2018-10-24

**Authors:** Wei Pan, Hui-wen Xu, Wen-ting Hao, Fen-fen Sun, Yan-fang Qin, Shan-shan Hao, Hua Liu, Jian-ping Cao, Yu-juan Shen, Kui-yang Zheng

**Affiliations:** 1 National Institute of Parasitic Diseases, Chinese Center for Disease Control and Prevention; Key Laboratory of Parasite and Vector Biology, Ministry of Health, Shanghai, China; 2 Jiangsu Key Laboratory of Immunity and Metabolism, Department of Pathogenic Biology and Immunology, Xuzhou Medical University, Xuzhou, Jiangsu Province China; 3 National Demonstration Center for Experimental Basic Medical Science Education (Xuzhou Medical University), Xuzhou, Jiangsu Province China; 4 Faculty of Clinical Medicine, Xuzhou Medical University, Xuzhou, Jiangsu Province China

**Keywords:** *Echinococcus granulosus* protoscoleces, Excretory-secretory products, B10 cells, TLR-2, PTEN, PI3K

## Abstract

**Background:**

Excretory-secretory products released by *Echinococcus granulosus* protoscoleces (EgPSC-ESPs) are well-known to regulate T cell responses. However, their direct influence on the differentiation of B cell subsets remains largely elusive. This study investigated the effects of EgPSC-ESPs on the differentiation of IL-10-producing B cells (B10), and explored the possible role of Toll-like receptor 2 (TLR-2) signaling in this process.

**Results:**

In comparison to phosphate buffered saline (PBS), B cells exposed to the excretory–secretory products (ESPs) generated higher percentages of B10 cells, with higher expression of IL-10 mRNA, and larger amount of IL-10 production, which were in a dose dependent way. The mRNA and protein expression of TLR-2 in the ESPs-stimulated B cells were significantly higher than those in PBS, which was consistent to the results in B cells isolated from EgPSC infected mice. Moreover, TLR-2^−/−^ B cells in response to ESPs stimulation expressed lower levels of IL-10 mRNA and produced undetectable IL-10 in comparison to those in normal B cells. In addition, Phosphatase and tensin homolog deleted on chromosome ten/AKT/Phosphatidylinositol-3 kinase (PTEN/AKT/PI3K) pathway was activated in ESPs-treated B cells, which was also dependent on TLR-2 signaling. Pam3CSK4, the agonist of TLR-2, could mock the effects of ESPs on the expression of PTEN, AKT and PI3K.

**Conclusion:**

Overall, this study revealed that TLR-2 signaling was required for B10 induction mediated by EgPSC-ESPs, which might be an immunomodulatory target against the parasite infection.

**Electronic supplementary material:**

The online version of this article (10.1186/s12865-018-0267-7) contains supplementary material, which is available to authorized users.

## Background

The genus of *Echinococcus* belongs to the family Taeniidae, and four species are recognized in the genus, namely *Echinococcus granulosus* (*E. granulosus*), *E. multilocularis*, *E. oligarthrus* and *E. vogeli* [1]. *E. granulosus* is a major species of great medical significance among them, which causes cystic echinococcosis and mainly distributes in areas of Central Asia, China, South America and Africa [2]. *E. granulosus* can infect hosts and go unnoticed for several decades, as it has evolved immune subversive strategies to evade host immune responses, thus maintaining persistent infection. Exploring those immunological mechanisms will be beneficial to develop novel strategies to prevent the disease. Several studies have pinpointed the ESPs of the parasite as strong immunoregulators, which had the ability to induce Th2 cells, as well as Th2-type cytokines like IL-4 and IL-10 [3]. Also, stimulation with adult derived ESPs could impair the maturation of dendritic cells (DCs) and promote the induction of regulatory T cells (Treg) [4]. In brief, these data suggested the well-known T cell response mediated by the ESPs.

However, the regulation of B cells response in *E. granulosus* infection is still largely unknown. B cells have been well established to negatively regulate immune responses in recent years, which were defined as regulatory B cells (Breg or B10 cells) [5]. They evoked a variety of IL-10-dependent regulatory effects, including downregulation of proinflammatory cytokines, induction of Treg cells and production of TGF-β [6–8]. The ability of B10 cells to regulate innate and adaptive immune responses made them an ideal therapeutic target for the treatment of many immune-related disorders [9–12]. Several studies have revealed that, B10 cells were induced in response to infection of parasites like *Leishmania major* and *Schistosoma* [13, 14]. Stimulation with ESPs of *Leishmania* led to IL-10 production by splenic B cells [15]. Hence, these studies implied that B10 cells were associated with parasite infection. In particular, B10 cells were found to be stimulated by glycoconjugates derived from EgPSC [16]. Moreover, our lab recently found the increased frequencies of B10 cells in EgPSC infected mice and EgPSC-ESPs significantly promoted the induction of B10 cells [17]. However, its underlying modulatory mechanism is not yet identified.

Toll like receptor (TLR) is a class of transmembrane pattern recognition receptors which recognized conserved microbial molecules and linked microbial recognition to activation of the TLR-expressing cells including T cells, B cells, macrophages and DCs [6]. TLR-2 is a widely expressed receptor among 12 or even more TLRs. Studies have demonstrated that activation of TLR-2 could enhance TLR-2-dependent IL-10 production from T cells and potentiate Treg cells generation [18]. DCs could also be activated through TLR-2 pathway, thus releasing more amounts of regulatory cytokines like IL-10 and TGF-β. Moreover, activated DCs polarized Th0 cells to Treg cells, highlighting TLR-2-dependent immunomodulatory function in DCs [19]. Therefore, TLR-2 plays crucial modulatory roles in both innate and adaptive immune response. Nevertheless, it is unclear whether TLR-2 exerts a role in the process of parasite-induced B10 differentiation. There was evidence showing that soluble egg antigens (SEA) from *Schistosoma mansoni* stimulated IL-10 production from B cells [20], and exclusively stimulated the upregulation of TLR-2 expression in B cells [21], suggesting a possible link between B10 and TLR-2 in parasite infection.

This study aimed to investigate the in vitro effects of EgPSC-ESPs on the differentiation of B10 cells and explore the role of TLR-2 in triggering the event. The results showed that EgPSC-ESPs induced the increase of IL-10 production and activated PTEN/AKT/PI3K pathway in B cells through TLR-2-dependent signaling. The current study demonstrated for the first time that TLR-2 was required for B10 induction promoted by EgPSC-ESPs, which presented a novel mechanism of immune evasion adopted by the parasite.

## Methods

### Mice, parasites, infection

Female C57BL/6 wild type mice and TLR-2^−/−^ mice were obtained from Shanghai Laboratory Animal Center (SLAC, Shanghai, China) and were bred in the Experimental Animal Center of Xuzhou Medical University. Eighteen C57BL/6 (wild type) mice and 18 TLR-2^−/−^ mice were sacrificed in the whole experiments. Each mouse was euthanized by cervical dislocation under intraperitoneal injection of 0.2 ml 4% sodium pentobarbital anesthesia solution. All animal procedures were approved by the Laboratory Animal Welfare and Ethics Committee (LAWEC) of Xuzhou Medical University, China (No. SCXK<SU> 2010–0003).

The EgPSC were obtained by puncturing the fertile sheep hydatid cysts under aseptic conditions according to the protocols detailed in Carmena et al. [22]. And the method for the establishment of PSC infected mice was mentioned in our previous studies [17, 23].

### EgPSC cultivation and ESPs collection

EgPSC were cultured and their ESPs were prepared as previously described [17, 24]. The concentration of collected ESPs was measured using the bicinchoninic acid (BCA) protein concentration assay kit (Beyotime Biotech, Beijing, China). The endotoxin in ESPs was carefully removed according to the protocol of Solution Endotoxin Erasol Kit (TIAN, Beijing, China) and the concentration of endotoxin was detected using Chromogenic End-point TAL Kit (TIANDZ, Beijing, China).

### B cell cultivation

CD19^+^B cells from the spleen of normal or infected C57BL/6 or TLR-2^−/−^ mice were sorted positively using a mouse CD19^+^B cell isolation kit (Miltenyi, Bergisch Gladbach, Germany); the cell purity was routinely > 90%. Purified B cells were cultured in 24-well plates (5 × 10^5^ cells/well) with or without EgPSC-ESPs (5 μg/ml), LPS (10 μg/ml) or TLR-2 agonist Pam3CSK4 (300 ng/ml). After 72 h of culture, cells and supernatants were collected for further analysis. Similarly, control or infected CD19^+^B cells were isolated for investigating on the expression of indicated genes both in mRNA and protein levels.

### Quantitative real-time PCR

RNA was extracted from cultured or freshly isolated B cells and the cDNA was synthesized for quantitative real-time PCR. The exact procedure for the PCR was described in the previous study [17]. The primers were listed in Table 1. All experiments were performed in triplicate and the relative expression of related genes was indicated by comparative cycling threshold (Ct) value normalized against an endogenous reference (GAPDH) using the 2^-△△Ct^ method.

**Table 1:** the real-time RT-PCR primers used in the study

Primer names	Sequences
IL-10	F: 5’-GCTCCAGAGCTGCGGACT-3’
	R: 5’-TGTTGTCCAGCTGGTCCTTT-3’
IL-6	F: 5’-CCACGGCCTTCCCTAC-3’
	R: 5’-AAGTGCATCATCGTTGT-3’
TNF-α	F: 5’-CATCTTCTCAAAATTCGAGTGACAA-3’
	R: 5’-TGGGAGTAGACAAGGTACAACCC-3’
TLR-2	F: 5’-TGTCTCCACAAGCGGGACTT-3’
	R: 5’-TTCGATGGAATCGATGATGTTG-3’
PTEN	F: 5’-AATTCCCAGTCAGAGGCGCTATGT-3’
	R: 5’-GATTGCAAGTTCCGCCACTGAACA-3’
PI3K	F: 5’-TCGGTCTGTAGATGAGGC-3’
	R: 5’-CGGAGGAATGGATGAGGG-3’
AKT	F: 5’-GTCGTCGCCAAGGATGAGG-3’
	R: 5’-GGTCGTGGGTCTGGAATGA-3’
TLR-9	F: 5’-TGGCATGGCTACCTTTGCTAG-3’
	R: 5’-AAATAGAGTCTTGCGGCTCCC-3’
GAPDH	F: 5’-CAACTTTGGCATTGTGGAAGG-3’
	R: 5’-ACACATTGGGGGTAGGAACAC-3’

### Flow cytometric analysis

Single-cell suspensions were prepared and filtered with a cell strainer. The staining was performed using the anti-CD19 antibody (clone eBio1D3) and anti-IL-10 antibody (clone JES5-16E3) or isotype control (from eBioscience, USA). For intracellular staining of IL-10, 50 ng/ml phorbolmyristate acetate (Sigma-Aldrich, USA), 500 ng/ml ionomycin (Sigma-Aldrich, USA), 10 μg/ml LPS (Sigma-Aldrich, USA), 10 μg/ml Brefeldin A (eBioscience,USA) and 2 μM monensin (eBioscience, USA) were added to the culture for the last 5 h before staining. The stained cells were determined by FACSCanto II flow cytometer (BD Biosciences, USA). Data were analyzed using the FlowJo software (version 7.2.5; Tree Star, Ashland, OR).

### Cytokine analysis

Mouse IL-10 ELISA Ready-SET-Go!® Kit (eBioscience, USA) was used to detect IL-10 levels in culture supernatants according to the recommendations of the manufacturer. The cytokine concentrations were calculated using standard curves.

### Western blot analysis

Total protein was extracted from freshly isolated or cultured B cells and the concentration was determined with the BCA protein concentration assay kit. Sample protein was separated by electrophoresis in 10% SDS-PAGE with the Bio-Rad electrophoresis system (Hercules, CA, USA). The primary antibodies (rabbit anti-PTEN, PI3K p85α, total-AKT1/2/3, p-AKT (ser473), TLR-2, TLR-4, Abcam, UK, 1:1000 dilutions) were incubated at 4 °C overnight. The secondary antibodies (anti-rabbit IgG, 1:2000 dilutions) were incubated for 2 h at room temperature. The membrane containing antibody-protein complexes were visualized with an enhanced chemiluminescence detection system on radiograph film (Bio-rad, Hercules, CA, USA). The bands were scanned and analyzed by the software Quantity ONE (Bio-rad, Hercules, CA, USA). The expression of protein in each sample was normalized by GAPDH.

### Statistical analysis

Data were expressed as means ± standard deviation (SD). Differences were analyzed by one-way ANOVA using SPSS 20.0 version; multiple comparisons between individual groups was performed using Bonferonni correction or Student-Newman-Keuls (S-N-K) method. *P* < 0.05 was considered to indicate statistical significance.

## Results

### The IL-10 production in B cells was directly triggered by EgPSC-ESPs in vitro

Previous studies have revealed that the glycoconjugates separated from somatic antigens of EgPSC stimulated IL-10 production in cultured B cells [16]. However, whether EgPSC-ESPs can trigger this process remains unclear. To exclude the disturbance of the endotoxin on the B differentiation, this study previously removed the endotoxin in the EgPSC-ESPs, and the concentration was lower than 0.015 EU/ml.

**Fig. 1:**
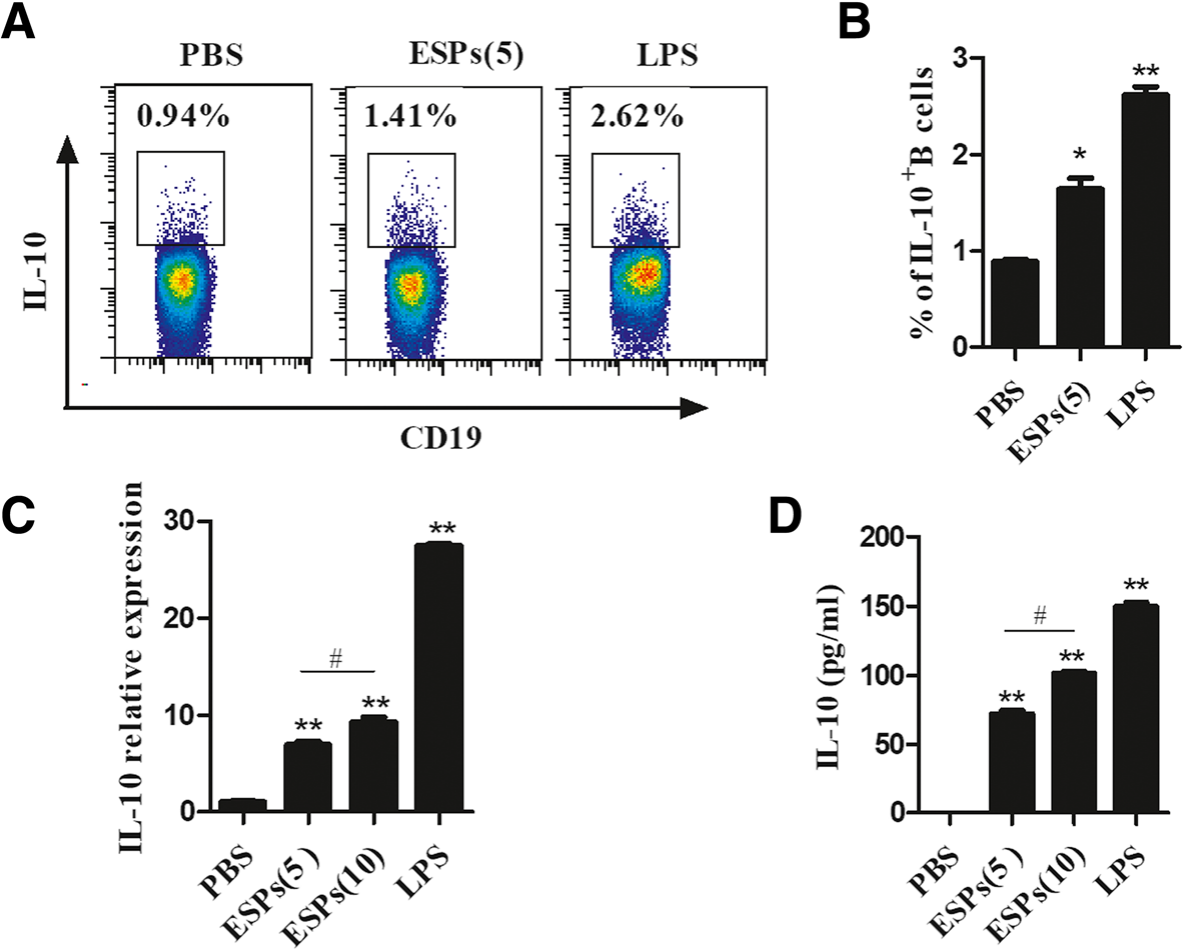
The direct induction of IL-10 secreting B cells by EgPSC-ESPs in vitro*.* Splenic CD19^+^B cells were sorted from and cultured for 72 h in the presence of PBS, EgPSC-ESPs (5, 10 μg/ml), or LPS (10 μg/ml). The cells or supernatants were collected for further analysis. **a** Representative flow cytometry plots of IL-10^+^CD19^+^B cells post stimuli. **b** The statistic results of flow cytometry. **c** The relative expression of IL-10 mRNA in B cells. **d** The IL-10 production in culture supernatants. Data are expressed as means ± SD of triplicate wells in one round experiment (*n* = 6), and the results were repeated in three independent experiments. Differences were analyzed by one-way ANOVA with Bonferroni correction. VS PBS group, *P < 0.05; **P < 0.001. ESPs(5) VS ESP(10), ^#^*P* < 0.05

To confirm the assumption, CD19^+^B cells were sorted from the splenic cells of normal C57BL/6 mice, and then cultured with PBS, EgPSC-ESPs (5, 10 μg/ml) or Lipopolysaccharide (LPS) (10 μg/ml) for 72 h, respectively. As shown in Fig. 1a and b, ESPs significantly increased the frequencies of IL-10^+^CD19^+^B cells compared with those in PBS group (*P* < 0.05). Moreover, the relative expression of IL-10 mRNA was significantly upregulated (*P* < 0.001, Fig. 1c), and the IL-10 production in the supernatants was significantly higher (*P* < 0.001, Fig. 1d), which were both in a dose dependent way. As a positive stimulant, LPS stimulated higher frequencies of B10, expression of IL-10 mRNA and IL-10 production in comparison to those in PBS and ESPs groups. These results showed EgPSC-ESPs could directly induce the differentiation of B10 cells in vitro.

### TLR-2 in B cells was significantly upregulated by EgPSC-ESPs

**Fig. 2:**
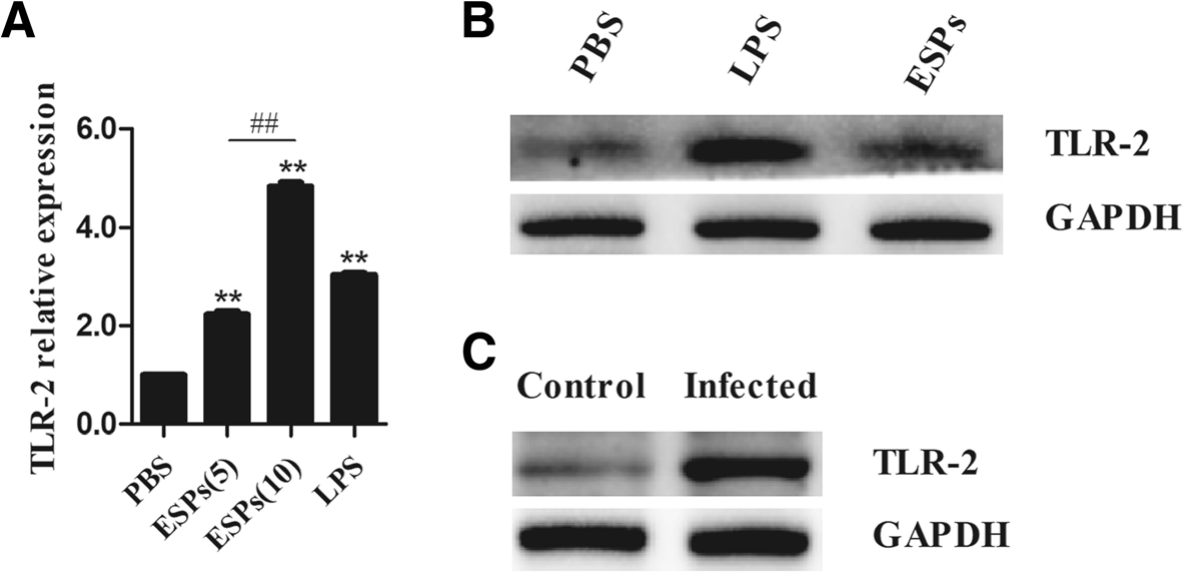
The expression of TLR-2 in B cells stimulated by EgPSC-ESPs or in B cells isolated from infected mice. **a** The mRNA levels of TLR-2 in B cells stimulated by PBS, EgPSC-ESPs (5, 10 μg/ml), or LPS (10 μg/ml). **b** The protein levels of TLR-2 in B cells post stimuli mentioned. **c** The expression of TLR-2 in B cells isolated from the spleen of control and PSC infected mice. The data represent the means ± SD of triplicate wells in one round experiment (*n* = 6), and the results were repeated in three independent experiments. Differences were analyzed by one-way ANOVA and Bonferonni correction. VS PBS group, *P < 0.05; **P < 0.001. ESPs(5) VS ESP(10), ^#^*P* < 0.05; ^##^*P* < 0.001

On account of the findings that TLR-2 is essential in the induction of regulatory DCs, Th2 and Treg cells [18, 19], this study speculated the association of the receptor in B10 differentiation mediated by the ESPs. To confirm the assumption, the changes of TLR-2 in cultured B cells were investigated and compared. As shown in Fig. 2a, the relative expression of TLR-2 mRNA in B cells cultured with ESPs were significantly upregulated in contrast with those in PBS-stimulated B cells (*P* < 0.001). Moreover, the change tendency of TLR-2 protein level was similar with their mRNA alternation (Fig. 2b). In addition, the B cells isolated from infected mice expressed a significantly higher levels of TLR-2 in compared to these in control mice (Fig. 2c), which was consistent to the changes of TLR-2 expression in ESPs stimulated B cells. These results showed that TLR-2 in B cells could be activated by the ESPs.

### TLR-2 was required for B10 induction mediated by EgPSC-ESPs

**Fig. 3:**
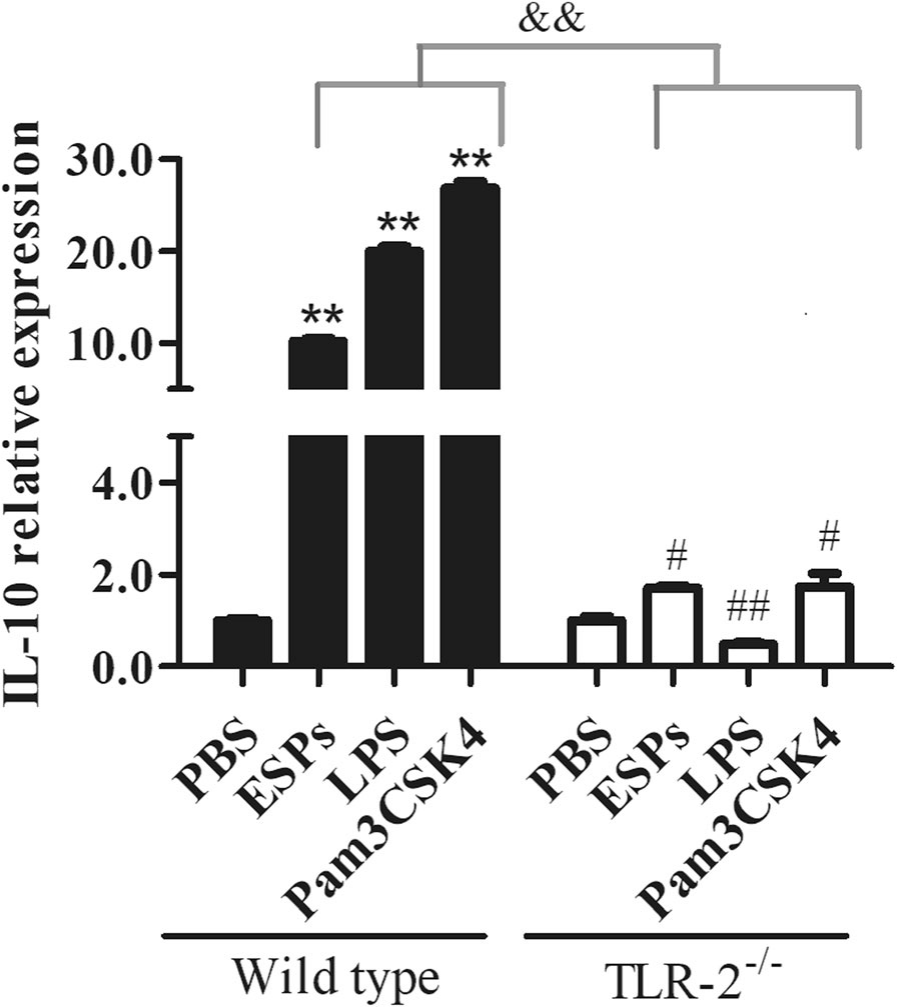
The comparison of IL-10 relative expression in wild type and TLR-2^−/−^ B cells stimulated by EgPSC-ESPs. CD19^+^B cells isolated from wild type and TLR2^−/−^ mice were cultured for 72 h in the presence of PBS, EgPSC-ESPs (5 μg/ml), LPS (10 μg/ml) or Pam3CSK4 (300 ng/ml). The relative expression of IL-10 in cultured B cells was compared. Data are expressed as means ± SD of triplicate wells in one round experiment (*n* = 6), and the results were repeated in three independent experiments. Differences were analyzed by one-way ANOVA or S-N-K method. VS PBS group in wild type B cells, ^**^P < 0.001; VS PBS group in TLR-2^−/−^ B cells, ^#^*P* < 0.05; ^##^*P* < 0.001. ^&&^*P* < 0.001, indicated the significant differences between EgPSC-ESPs, LPS and Pam3CSK4 (except for PBS) in wild type B cells and in TLR-2^−/−^ B cells

A previous study has suggested the association between TLR-2 and B10 in parasite infection [21]. This study assumed that the ESPs participated in the induction of B10 cells via TLR2 signaling in a direct manner. The B cells were sorted from the splenic cells of wild type or TLR-2^−/−^ mice, and then cultivated with PBS, EgPSC-ESPs (5 μg/ml), LPS (10 μg/ml) or Pam3CSK4 (300 ng/ml) for 72 h. Their effects on B10 induction were compared. As shown in Fig. 3, the relative expression of IL-10 in TLR-2^−/−^ B cells were significantly lower than wild type B cells when these cells were dealt with stimulation of ESPs, LPS and Pam3CSK4, respectively (*P* < 0.001). Moreover, the IL-10 production in TLR-2^−/−^ B cells post various stimuli became undetectable (data not shown). The results suggested that similar to the Pam3CSK4, TLR-2 was also required for B10 induction mediated by the ESPs.

### The PTEN/AKT/PI3K pathway was activated in EgPSC-ESPs stimulated B cells via TLR-2 signaling

PI3K/AKT is a cellular metabolic pathway, which is in correlation with cell proliferation, apoptosis, and tumor genesis. As a suppressor of PI3K pathway, PTEN antagonizes PI3K activation thus inhibiting inflammatory response. However, whether PTEN/PI3K pathway was associated within the induction of B10 cells triggered by the ESPs has not been established. This study therefore detected the expression changes of PTEN, PI3K and AKT in cultivated B cells exposed to the ESPs.

**Fig. 4:**
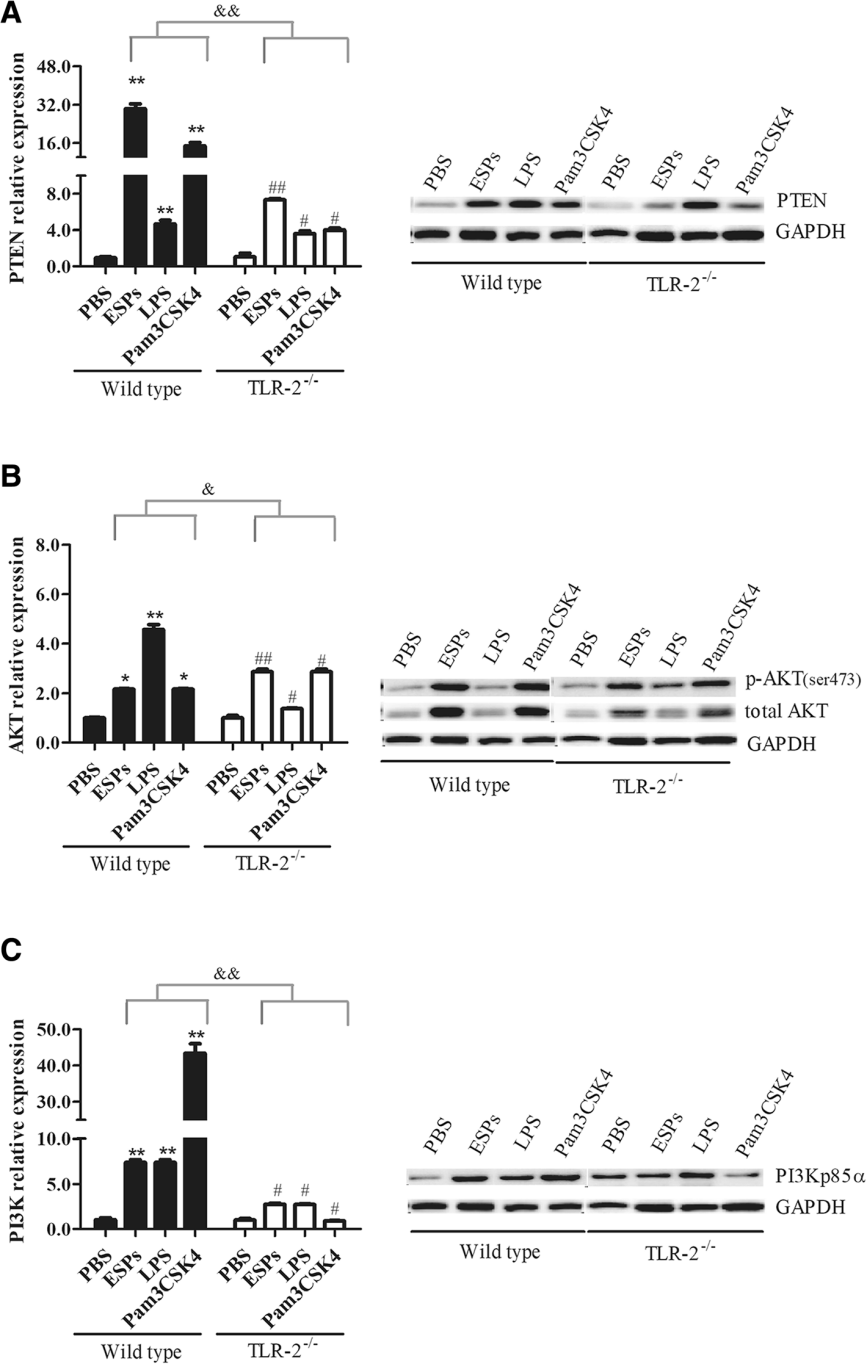
The effects of EgPSC-ESPs on the expression of PTEN, AKT and PI3K in wild type and TLR-2^−/−^ B cells. The sorted B cells were stimulated similarly with Fig. 3. **a**–**c** showed the mRNA levels of PTEN, AKT, and PI3K in cultured B cells analyzed by RT-PCR (left part), and their related protein expressions were analyzed by western blot (right part), respectively. Each western blot experiment used the same GAPDH as control for all detected proteins. Data are expressed as means ± SD of triplicate wells in one round experiment (*n* = 6), and the results were repeated in three independent experiments. Differences were analyzed by one-way ANOVA and S-N-K method. VS PBS group in wild type B cells, ^*^P < 0.05; ^**^P < 0.001; VS PBS group in TLR-2^−/−^ B cells, ^#^*P* < 0.05; ^##^*P* < 0.001. ^&^*P* < 0.05, ^&&^*P* < 0.001, indicated the significant differences between EgPSC-ESPs, LPS and Pam3CSK4 (except for PBS) in wild type B cells and in TLR-2^−/−^ B cells

As shown in Fig. 4a (left), the mRNA of PTEN in wild type mice-derived B cells after stimulation of ESPs was expressed significantly higher than those in PBS groups (*P* < 0.001). Moreover, PTEN expression became significantly lower in TLR-2^−/−^ B cells in comparison to those in wild type B cells (*P* < 0.001), although the expression tendency was similar. In protein level, PTEN expression in wild type B cells exposed to ESPs and Pam3CSK4 was significantly higher than those in PBS groups, however, their expression become much lower in TLR-2^−/−^ B cells (Fig. 4a, right). Thereby, these results suggested that PTEN in B cells was regulated by the ESPs in TLR-2 dependent manner.

The expression of AKT in B cells was also determined. As shown in Fig. 4b left, ESPs and Pam3CSK4 significantly upregulated the mRNA of AKT in wild type B cells (*P* < 0.05), and the tendency was similar in TLR-2^−/−^ B cells. Fig. 4b (right) showed the AKT expression in protein level. ESPs and Pam3CSK4 stimulated higher levels of total AKT and p-AKT (ser473) in wild type B cells in comparison to PBS and LPS group. The expression of PI3K was shown in Fig. 4c. ESPs and Pam3CSK4 showed obvious effect on its expression both in mRNA and protein levels in wild type B cells. However, the expression of PI3K p85a was slightly lower in TLR-2^−/−^ B cells.

## Discussion

Unlike T cells, the regulation of B cell response in EgPSC infection is still largely unknown. A previous study has found that glycoconjugates isolated from EgPSC somatic antigens stimulated the secretion of IL-10 by B cells [16]. This study complementarily showed that the ESPs of EgPSC significantly induced the differentiation of B10 cells. Moreover, TLR-2 signaling was shown to be required for the induction.

B10 cells serve as negative regulator of immune responses solely attributed to IL-10 production in B cells [9–12], which have been found to dampen T-cell-mediated inflammation, regulate the autoimmunity and maintain immune system homeostasis [25–27]. There is accumulating evidence showed that the percentages of B10 cells were expanded in hosts following parasite infection [13–15, 28–30], which suggests that the generation of B10 cells is one of common immune escape mechanisms adopted by parasites. This study found the B10 was induced directly by EgPSC derived-ESPs in vitro, which partly contributed to the increase frequencies of B10 in infected mice [17] and suggested that induction of B10 was another way to mediate the immune escape in *E. granulosus* infection.

Given the well-known fact, that LPS stimulated IL-10 production by naïve B cells via TLR signaling (TLR-2, TLR-4, MyD88) [6], these related receptors were therefore speculated to be associated in the induction of B10 by EgPSC-ESPs. To exclude the disturbance of LPS, this study previously removed the endotoxin in the ESPs, and the endotoxin level was lower than 0.015 EU/ml. However, they still significantly promoted the induction, which suggested the induction should not be mediated by LPS in the ESPs. It is possible that ES-62-like molecule that has shown to restore the levels of B10 cells, may also exist in the EgPSC-ESPs, thereby mediating the B cell response [31].

There were accumulating evidences showing the essential role of TLR-2 in mediating the generation of B cells, DCs and Treg cells [6, 32, 33]. Particularly, the SEA of *Schistosoma mansoni* was reported to exclusively stimulate the TLR-2 expression in B cells [21]. This study confirmed that the upregulated expression of TLR-2 was required for B10 induction mediated by EgPSC-ESPs. Not only that, this study also provided some clues for investigating the underlying mechanism in the induction process.

The gene expression of MyD88 in wild-type B cells exposed to the ESPs was significantly increased while decreased in TLR-2^−/−^ B cells (data not shown), which showed that MyD88 was partly activated by TLR-2. Previous study has indicated that MyD88 was required in LPS stimulated IL-10 production by B cells [6]. Thus TLR-2/MyD88 pathway should be associated in triggering B10 induction. Besides, TLR-4 and TLR-9 were shown to play an essential role in the induction process [6, 34]. This study also found the altered expressions of the 2 TLRs both in *the vitro* B cell culture system and in the B cells isolated from infected mice (shown in Additional file 1: Figure S1). In addition, IL-35, the novel regulator of B10 cells [35–37], should also be considered to elucidate the generation mechanism of B10 cells.

The PTEN/AKT/PI3K pathway in B cells was activated after exposure to the ESPs. PTEN is a PI3-K pathway suppressor, a phosphoinositide-3-phosphatase to produce phosphatidylinositol4,5-bisphosphate (PI(4,5)P2). By generating PI(4,5)P2, PTEN antagonized the PI3K/AKT-dependent cell signaling, thus suppressing PI3K/AKT mediated cellular activities, such as cell proliferation, apoptosis, and tumorigenesis [38]. Moreover, most recently PTEN/PI3K has been found to be a central signal transduction axis mediating B cell homeostasis and influencing B cell functional responses [39, 40]. Notably, the defective PTEN regulation was reported to contribute to B cell hyperresponsiveness in systemic lupus erythematosus (SLE) [41]. In addition, over-expression of PTEN could inhibit LPS-induced pulmonary fibrosis [42]. The present study found the dramatic increase of PTEN expression in B cells when cultivating with ESPs, which in combination with other studies emphasized the role of PTEN in the differentiation of B10 cells. However, the expression of PTEN was significantly lower in TLR-2^−/−^ B cells, suggesting the requirement of TLR-2 signaling. Collectively, these results implied that TLR-2/PTEN/PI3K/AKT pathway was activated in the induction of B10 cells.

Interestingly, this study also found that EgPSC-ESPs can regulate the expression of IL-6 and TNF-α in B cells, which also seems to be dependent on the TLR-2 signaling (shown in Additional file 2: Figure S2). Our previous study has showed that the expansion of B10 cells were dependent on the carbohydrates in the ESPs [17]. In addition, the E4(+) (a glycoconjugate-enriched fraction from EgPSC) stimulated the secretion of a high concentration of IL-6, followed by IL-10 and TNF-a by normal peritoneal B cells [16], which were consistent to the results of the present study. These results suggested that EgPSC-ESPs might induce a mixted pro-inflammatory and anti-inflammatory response in B cells, thereby maintaining immunity homeostasis. This seems to be expected, because the ESPs contained diverse components that perform different biological functions [43].

## Conclusion

In conclusion, this study showed that EgPSC-ESPs directly triggered B cells to secrete IL-10 in vitro, which required TLR-2 signaling while PTEN/AKT/PI3K pathway was activated. Overall, this study supported the role of EgPSC-ESPs in promoting the induction of B10 cells in TLR-2 dependent manner, and future work should pay more attention to elucidate the induction mechanism of B10 cells in *E. granulosus* infection.

## Additional files

Additional file 1: **Figure S1.** The expression of TLR-4 and TLR-9 in B cells stimulated by EgPSC-ESPs or in B cells isolated from infected mice. (A) The protein levels of TLR-4 in B cells stimulated by PBS, EgPSC-ESPs (5 μg/ml), or LPS (10 μg/ml). (B) The mRNA expression of TLR-9 in B cells post stimuli mentioned. Differences were analyzed by one-way ANOVA. VS PBS group, *P < 0.05; **P < 0.001. (TIF 814 kb)

Additional file 2: **Figure S2.** The comparison of IL-6 and TNF-α relative expression in wild type and TLR-2^−/−^ B cells stimulated by EgPSC-ESPs. CD19^+^B cells isolated from wild type and TLR2^−/−^ mice were cultured for 72 h in the presence of PBS, EgPSC-ESPs (5 μg/ml), LPS (10 μg/ml) or Pam3CSK4 (300 ng/ml). The relative expression of IL-6 and TNF-α in cultured B cells was compared. Data are expressed as means ± SD of triplicate wells in one round experiment (*n* = 6). Differences were analyzed by one-way ANOVA or S-N-K method. VS PBS group in wild type B cells, ^*^P < 0.05; ^**^P < 0.001; VS PBS group in TLR-2^−/−^ B cells, ^#^*P* < 0.05; ^##^*P* < 0.001. ^&^*P* < 0.05, ^&&^*P* < 0.001, indicated the significant differences between EgPSC-ESPs, LPS and Pam3CSK4 (except for PBS) in wild type B cells and in TLR-2^−/−^ B cells. (TIF 360 kb)

## Abbreviations

B10: IL-10-producing B cells
Breg: Regulatory B cells
DCs: Dendritic cells
EgPSC-ESPs: *Echinococcus granulosus* protoscoleces
ESPs: Excretory–secretory products
LPS: Lipopolysaccharide
PBS: Phosphate buffered saline
PI(4,5)P2: Phosphatidylinositol4,5-bisphosphate
PI3K: Phosphatidylinositol-3 kinase
PTEN: Phosphatase and tensin homolog deleted on chromosome ten
SEA: Soluble egg antigens
SLE: Systemic lupus erythematosus
TLR: Toll like receptor
TLR-2: Toll-like receptor 2
TLR-4: Toll-like receptor 4
TLR-9: Toll-like receptor 9
Treg: Regulatory T cells
