# Supplementary figures and images for: The excretory-secretory products of Echinococcus granulosus protoscoleces stimulated IL-10 production in B cells via TLR-2 signaling
Source: BMC Immunol. 2018 Oct 24;19:29. doi: 10.1186/s12865-018-0267-7 (PMC6201587; doi:10.1186/s12865-018-0267-7)

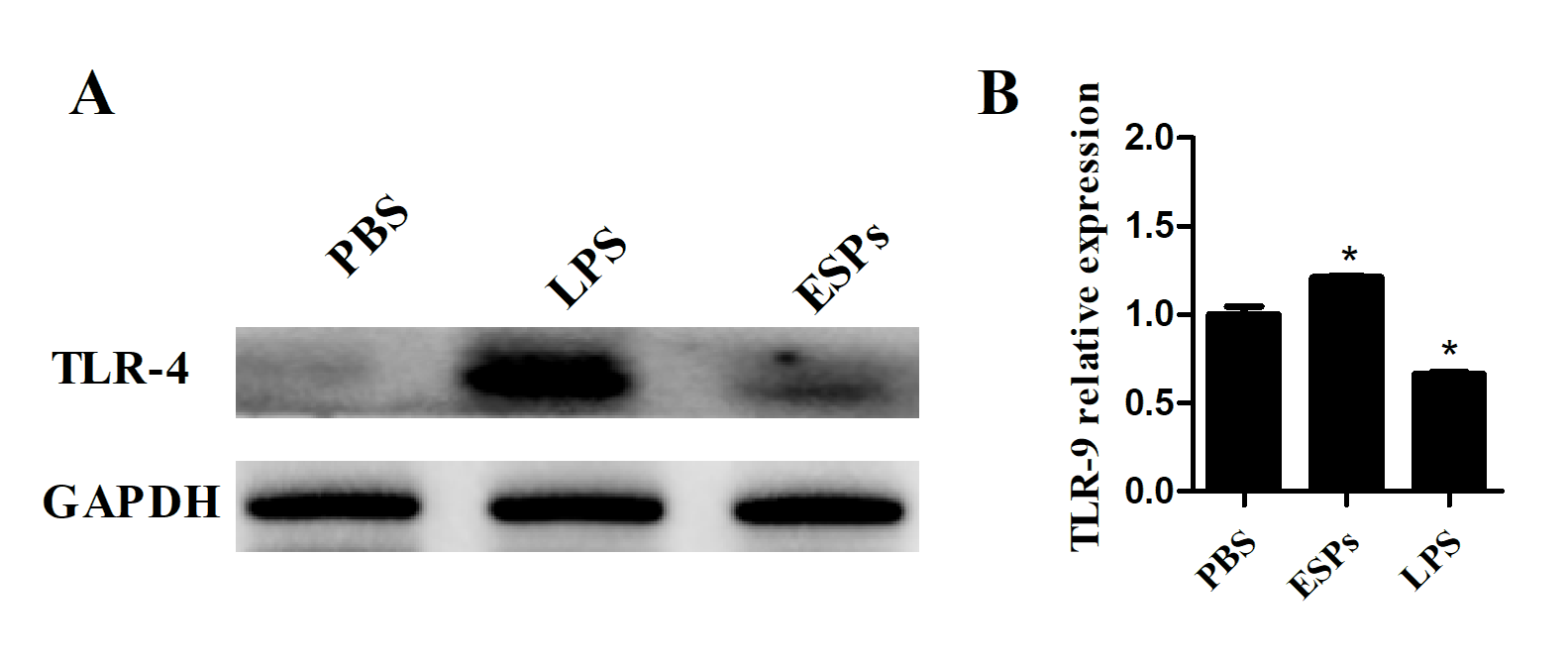

Supplement: Supplementary file 1 — Figure S1. The expression of TLR-4 and TLR-9 in B cells stimulated by EgPSC-ESPs or in B cells isolated from infected mice. (A) The protein levels of TLR-4 in B cells stimulated by PBS, EgPSC-ESPs (5 μg/ml), or LPS (10 μg/ml). (B) The mRNA expression of TLR-9 in B cells post stimuli mentioned. Differences were analyzed by one-way ANOVA. VS PBS group, *P < 0.05; **P < 0.001. (TIF 814 kb) [file 12865_2018_267_MOESM1_ESM.tif]

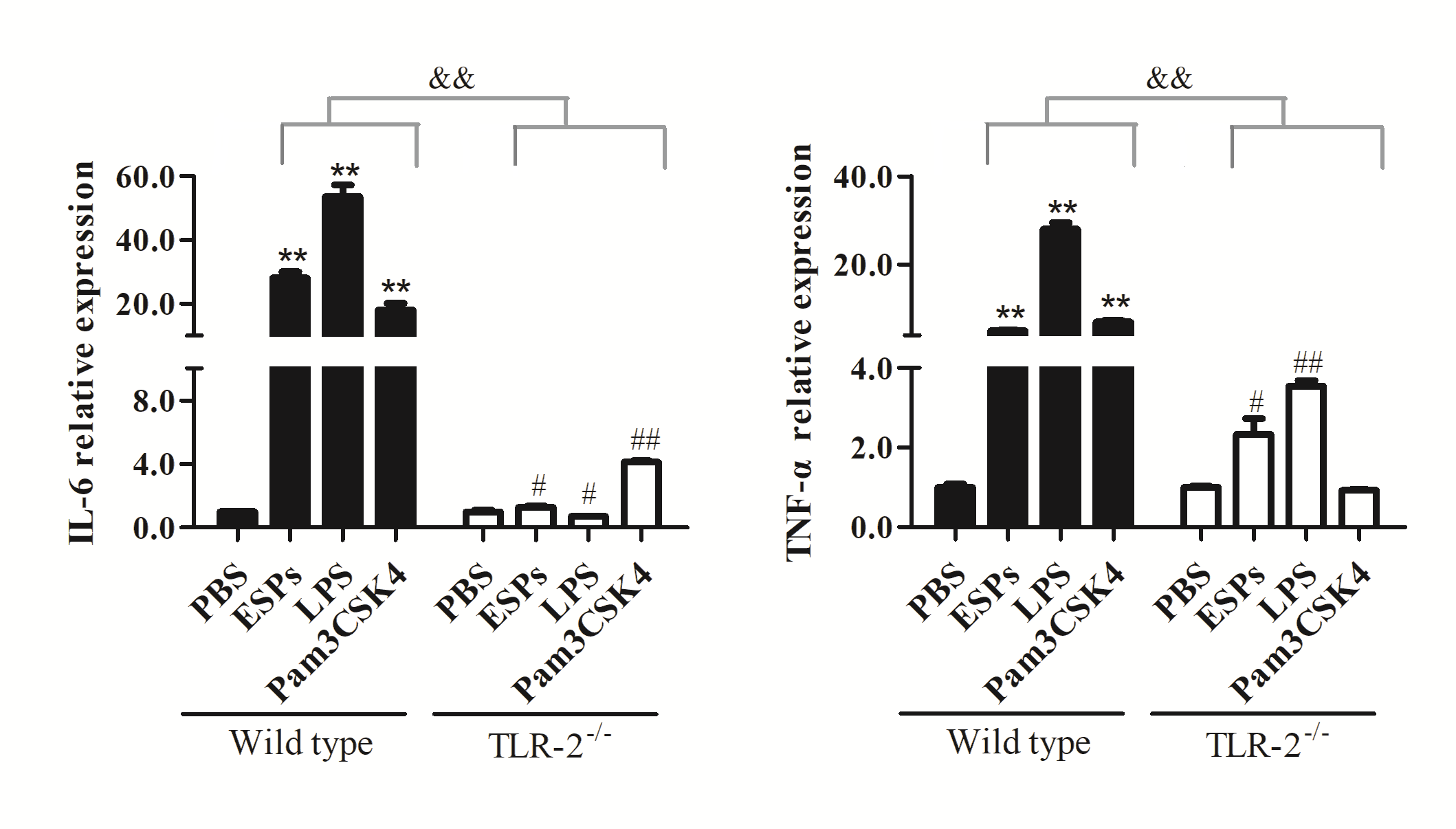

Supplement: Supplementary file 2 — Figure S2. The comparison of IL-6 and TNF-α relative expression in wild type and TLR-2−/− B cells stimulated by EgPSC-ESPs. CD19+B cells isolated from wild type and TLR2−/− mice were cultured for 72 h in the presence of PBS, EgPSC-ESPs (5 μg/ml), LPS (10 μg/ml) or Pam3CSK4 (300 ng/ml). The relative expression of IL-6 and TNF-α in cultured B cells was compared. Data are expressed as means ± SD of triplicate wells in one round experiment (n = 6). Differences were analyzed by one-way ANOVA or S-N-K method. VS PBS group in wild type B cells, *P < 0.05; **P < 0.001; VS PBS group in TLR-2−/− B cells, #P < 0.05; ##P < 0.001. &P < 0.05, &&P < 0.001, indicated the significant differences between EgPSC-ESPs, LPS and Pam3CSK4 (except for PBS) in wild type B cells and in TLR-2−/− B cells. (TIF 360 kb) [file 12865_2018_267_MOESM2_ESM.tif]
